# Investigation of the allergens in 2,316 children with allergic rhinitis from Guangdong, China

**DOI:** 10.3389/fped.2022.1051993

**Published:** 2022-11-24

**Authors:** Nannan Zhang, Yunwen Wu, Zequn Wei, Jinen Li, Jiao Shi, Rong Cai, Hailing Huang, Siyuan Ouyang, Qingfeng Zhang

**Affiliations:** ^1^ Department of Otorhinolaryngology, Head and Neck Surgery, Shenzhen University General Hospital, Shenzhen, China; ^2^ Department of Otorhinolaryngology, Head and Neck Surgery, Huizhou Third People's Hospital, Huizhou, China; ^3^ Shenzhen University, Shenzhen, China

**Keywords:** allergenic rhinitis, children, allergens, specific IgE, epidemiology

## Abstract

Allergic rhinitis (AR) is one of the popular childhood diseases, bringing physical and metal burdens to the children and their families. The study was performed to detect common allergens eliciting AR in children, to investigate the prevalence of allergens in different age and gender cohorts, and to provide a reliable basis for clinical prevention and treatment of AR during childhood. We measured serum-specific IgE and performed inhalant and ingestion allergen examinations in 2,316 children with AR, in collaboration with BioSciTec GmbH. The prevalence of different allergens was determined according to gender, age, severity, and season. Among the 2,316 AR cases, the top five inhalant allergens were *Dermatophagoides pteronyssinus* (1,674 cases, 72.3%), *Dermatophagoides farinae* (1,520 cases, 65.6%), *Blomia tropicalis* (1,477 cases, 63.8%), Cockroach (602 cases, 26.0%), and Dog hair (602 cases, 26.0%). The top five ingestive allergens were Milk (1,111 cases, 48.0%), Egg white (543 cases, 23.4%), Shrimp/Crab (425 cases, 18.4%), Beef/Mutton (422 cases, 18.2%), and Egg yold (329 cases, 14.2%). AR severity analyses showed that 50.9% (1,180 cases) of *D. pteronyssinus* allergies were above level three, 47.9% (1,109 cases) of *D. farinae* allergies were above level three, only 23.3% (539 cases) of *B. tropicalis* allergies were level three, and *B. tropicalis* allergies were mainly of level 2. Other AR-inducing allergens mainly produced level one or two reactions. Regarding ingestion allergens, 7.9% (183 cases) of milk allergies and 4.7% (108 cases) of Shrimp/Crab allergies were above level three, and other allergens induced AR mainly of level one or two. The study investigated the major allergens eliciting AR in children from Guangdong, China, assessed the prevalence and severity among cohorts regarding age, gender, and season, and produced essential information on childhood AR, laying important references for AR prevention and treatment in the future.

## Introduction

Allergic diseases constitute a series of reactions of the immune system to allergens. Such reactions greatly reduce the life quality of patients and sometimes are life-threatening (1). Allergic rhinitis (AR) is a common form of allergy, which is a non-infectious chronic inflammatory disease of the nasal mucosa mediated by immunoglobulin E (IgE) due to exposure to allergens (2).

AR is an allergic disease with a globally increasing trend, and it is estimated that there are more than 500 million AR patients worldwide (3), thus constituting a substantial social and economic burden. Additionally, AR is the most common allergic disease in children, with a prevalence of approximately 3%–38%, respectively (4, 5). Hence, identifying allergens in children with AR plays is key for effective treatment and prevention schemes (6). Inhalation allergens are the main cause of AR, with typical clinical symptoms including sneezing attacks, runny nose, nasal itching, and nasal congestion. Ingestion allergens are a further main cause of AR in children, and recent studies have shown that ingestion allergens in childhood are associated with AR during adulthood (7). Serum-specific IgE determination is a basic method for detecting allergens in AR patients, which provides valuable evidence for AR diagnosis and individual-specific immunotherapy. Identifying the factors that contribute to allergic reactions is essential to improve public health, and determining the distribution of allergens is key to understanding such afflictions (8, 9). However, allergens vary widely, depending on location, climate, and lifestyle, with variability occurring even between different regions of the same country (10–15). A previous study suggested that the prevalence of AR has increased in adults and children in China in the past 20 years, which may be attributed to “Western” lifestyle, industrialization, and air pollution (16). China has a large population and covers a vast territory. Differences in climate, environment, dietary habits, living conditions, and economic development between regions may affect the effects of potential allergens, in addition to differences between genders and age classes. However, the main allergen sources, respective AR severities, and seasonal effects in children from southern China remain to be revealed.

In this study, we retrospectively analyzed 2,316 children with AR who resided in Shenzhen, Guangdong, China, between April 2019 and March 2022, and we explored sensitization differences between genders, age classes, and seasons. The results of the present study provide a clinical basis for scientific research and clinical prevention and treatment of AR in children.

## Materials and methods

### Ethics

This study was reviewed and approved by the institutional research ethics committee of Shenzhen University (NO:M202200341). Written informed consent was waived by the Institutional Review Board. All analyses adhered to the institutional guidelines and requirements of the Ethics Committee.

### Ar participant enrollment

A total of 2,316 patients showing AR clinical symptoms of AR were examined in Shenzhen University General Hospital from April 2019 to March 2022, including 1,480 male and 836 female children aged 1–14 years (mean age 7.61 ± 3.03 years). I)All patients should satisfy at least two of the following clinical symptoms: paroxysmal sneezing, runny nose, nasal itching, nasal congestion, and a history of sudden and recurrent episodes with a daily duration of more than 1 h; Physical signs: pale nasal mucosa, edema, abundant watery secretion, turbinate enlargement, allergy-associated black eye circles, and allergic folds in severe AR children; and at least one allergen was positive in a serum-specific IgE test. All patients had lived in Shenzhen for at least one year and had no history of long-term foreign travel.

### Clinical examination and data collection

Serum specific IgE (sIgE) concentrations of inhaled and ingested allergens were measured using a serum specific IgE assay (AllergyScreen, Mediwiss-Analytic GmbH, Moers, Germany). Serum specific IgE values in response to 18 inhalable allergens (*D.pteronyssinus, D.farinae, B. tropicalis,* cat hair, etc.) and ingested allergens (Egg white, Milk, Beef, Mango, Cashew nut, Shrimp, Crab, etc.) were detected. Responses to allergen concentrations >0.3 5IU/ml were considered positive. Moreover, severities were divided into six levels according to the concentration of allergens (Table 1).

**Table 1:** Determination of allergen results.

Severity	Reference range of sIgE concentration (IU/ml)	Outcome
6	>100.00	extremely high
5	50.00–100.00	very high
4	17.50–49.90	high
3	3.50–17.49	increased
2	0.70–3.49	Medium
1	0.35–0.69	Low
0	<0.35	none (undetectable)

### Statistical analyses

SPSS Statistics 26.0 (IBM, Armonk, NY, USA) was used to analyze the data. Count data were presented as an example (%), and comparison between groups was performed using a *χ*^2^ test. *P* < 0.05 was considered statistically significant.

## Results

### Distribution of allergens

**Figure 1:**
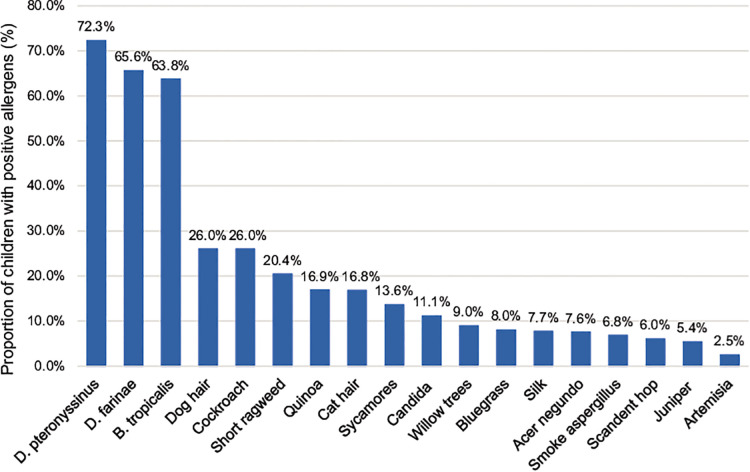
Distribution of inhaled allergens in 2,316 children with AR. The abscissa represents different inhaled allergens, and the ordinate exhibits their distributions in the AR children.

**Figure 2:**
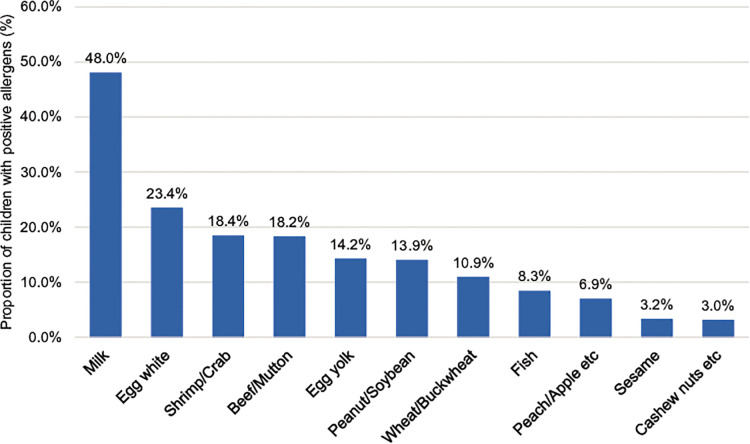
Distribution of ingestive allergens in 2,316 children with AR. The abscissa represents different ingestive allergens, and the ordinate exhibits their distributions in the AR children.

All 2,316 children with AR, aged 1–14 years and living in Shenzhen, were positive for at least one allergen. Among them, 1,337 were positive for more than five allergens (inhalation and food), 336 were positive for four allergens, 337 were positive for three allergens, 222 were positive for two allergens, and 84 were positive for one allergen. The distribution of various inhalation allergens is shown in Figure 1, among which the top five most common types of inhalation allergens were *D. pteronyssinus* (1,674 cases, 72.3%), *D. farinae* (1,520 cases, 65.6%), *B. tropicalis* (1,477 cases, 63.8%), cockroach (602 cases, 26.0%), and dog hair (602 cases, 26.0%). The distribution of various ingestion allergens is shown in Figure 2. The top five most common ingestion allergens were milk (1,111 cases, 48.0%), egg white (543 cases, 23.4%), shrimp/crab (425 cases, 18.4%), beef/lamb (422 cases, 18.2%), and Egg yolk (329 cases, 14.2%) (Figures 1, 2).

### Differences between male and female participants

Among 2,316 children with AR and aged 1–14 years, the top five common inhalation allergens in 1,480 male AR children were *D. pteronyssinus*, *D. farinae*, *B. tropicalis*, cockroach, and dog hair. The top five common ingestion allergens were milk, egg white, shrimp/crab, beef/mutton, and egg yolk. The top five common inhalation allergens in 836 female AR children were *D. pteronyssinus*, *B. tropicalis*, *D. farinae*, cockroach and dog hair. The top five common ingestion allergens were milk, egg white, beef/lamb, shrimp/crab, and egg yolk. There were significant differences between genders in the positive rates regarding *D. pteronyssinus* (*P* = 0.003), *D. farinae* (*P* = 0.000), *B. tropicalis* (*P* = 0.002), milk (*P* = 0.019), and shrimp/crab (*P* = 0.005) among different genders, whereas no significant differences in the positive rates of inhaled allergens and ingested allergens were observed between other cohorts (Table 2).

**Table 2:** Distribution of allergens in AR children of different genders and different ages.

Allergen	Total *n* = 2,316	Genders				Ages				
		Male *n* = 1,480 (%)	Female *n* = 836 (%)	χ2	*P* (*P* < 0.05)	1–3 *n* = 102 (%)	3–7 *n* = 988 (%)	7–14 *n* = 1,226 (%)	*χ* ^2^	*P* (*P* < 0.05)
*D. pteronyssinus*	1,674 (72.3%)	1,101 (74.4%)	573 (68.5%)	9.129	0.003	48 (47.1%)a	627 (63.5%)b	999 (81.5%)c	122.571	0
*D. farinae*	1,520 (65.6%)	1,010 (68.2%)	510 (61%)	12.409	0	35 (34.3%)a	542 (54.9%)b	943 (76.9%)c	164.407	0
*B. tropicalis*	1,477 (63.8%)	962 (65%)	515 (61.6%)	2.669	0.102	40 (39.2%)a	568 (57.5%)b	869 (70.9%)c	70.319	0
Cat hai	389 (16.8%)	252 (17%)	137 (16.4%)	0.184	0.668	26 (25.5%)a	143 (14.5%)b	220 (17.9%)a,b	10.487	0.005
Dog hair	602 (26%)	389 (26.3%)	213 (25.5%)	0.18	0.671	29 (28.4%)	252 (25.5%)	321 (26.2%)	0.46	0.795
Cockroach	602 (26%)	389 (26.3%)	213 (25.5%)	0.18	0.671	18 (17.6%)	267 (27%)	317 (25.9%)	4.251	0.119
Silk	178 (7.7%)	113 (7.6%)	65 (7.8%)	0.015	0.903	5 (4.9%)	66 (6.7%)	107 (8.7%)	4.398	0.111
Short ragweed	473 (20.4%)	291 (19.7%)	182 (21.8%)	1.461	0.227	19 (18.6%)	224 (22.7%)	230 (18.8%)	5.363	0.068
Artemisia pollen	58 (2.5%)	34 (2.3%)	24 (2.9%)	0.72	0.396	0 (0%)	30 (3%)	28 (2.3%)	4.01	0.135
Scandent hop	140 (6%)	72 (4.9%)	68 (8.1%)	10.053	0.002	6 (5.9%)	68 (6.9%)	66 (5.4%)	2.17	0.338
Quinoa	392 (16.9%)	251 (17%)	141 (16.9%)	0.003	0.954	12 (11.8%)a	188 (19%)a	192 (15.7%)a	6.434	0.04
Juniper	125 (5.4%)	70 (4.7%)	55 (6.6%)	3.578	0.059	7 (6.9%)	53 (5.4%)	65 (5.3%)	0.453	0.797
Sycamores	316 (13.6%)	196 (13.2%)	120 (14.4%)	0.559	0.454	10 (9.8%)	140 (14.2%)	166 (13.5%)	1.52	0.468
Willow trees	208 (9%)	141 (9.5%)	67 (8%)	1.495	0.221	7 (6.9%)	84 (8.5%)	117 (9.5%)	1.311	0.519
Bluegrass	186 (8%)	123 (8.3%)	63 (7.5%)	0.434	0.51	5 (4.9%)	75 (7.6%)	106 (8.6%)	2.239	0.326
Acer negundo	175 (7.6%)	106 (7.2%)	69 (8.3%)	0.911	0.34	4 (3.9%)	69 (7%)	102 (8.3%)	3.416	0.181
Smoke spergillus	158 (6.8%)	103 (7%)	55 (6.6%)	0.122	0.727	8 (7.8%)a,b	48 (4.9%)b	102 (8.3%)a	10.487	0.005
Candida	258 (11.1%)	152 (10.3%)	106 (12.7%)	3.132	0.077	10 (9.8%)a,b	83 (8.4%)b	165 (13.5%)a	14.33	0.001
Egg yolk	329 (14.2%)	225 (15.2%)	104 (12.4%)	3.345	0.067	18 (17.6%)	145 (14.7%)	166 (13.5%)	1.616	0.446
Egg white	543 (23.4%)	346 (23.4%)	197 (23.6%)	0.01	0.919	37 (36.3%)a	292 (29.6%)a	214 (17.5%)b	54.408	0
Milk	1,111 (48%)	737 (49.8%)	374 (44.7%)	5.481	0.019	56 (54.9%)a,b	518 (52.4%)b	537 (43.8%)a	18.372	0
Peanut/soybean	322 (13.9%)	205 (13.9%)	117 (14%)	0.009	0.923	14 (13.7%)	144 (14.6%)	164 (13.4%)	0.659	0.719
Sesame	75 (3.2%)	44 (3%)	31 (3.7%)	0.921	0.337	4 (3.9%)	30 (3%)	41 (3.3%)	0.324	0.85
Wheat/Buckwheat	252 (10.9%)	157 (10.6%)	95 (11.4%)	0.314	0.575	11 (10.8%)	111 (11.2%)	130 (10.6%)	0.226	0.893
Cashew nuts	70 (3%)	50 (3.4%)	20 (2.4%)	1.772	0.183	1 (1%)	32 (3.2%)	37 (3%)	1.609	0.447
Beef/Mutton	422 (18.2%)	287 (19.4%)	135 (16.1%)	3.772	0.052	22 (21.6%)	178 (18%)	222 (18.1%)	0.805	0.668
Fish	193 (8.3%)	123 (8.3%)	70 (8.4%)	0.003	0.958	8 (7.8%)	89 (9%)	96 (7.8%)	1.027	0.598
Shrimp/Crab	425 (18.4%)	297 (20.1%)	128 (15.3%)	8.067	0.005	21 (20.6%)	185 (18.7%)	219 (17.9%)	0.628	0.731
Peach/Apple	160 (6.9%)	105 (7.1%)	55 (6.6%)	0.221	0.638	6 (5.9%)	63 (6.4%)	91 (7.4%)	1.105	0.575

### Distribution of allergens in different age groups

The distribution of positive inhalation allergens and ingestion allergens in children of different ages is shown in Table 2. The children were divided into three age groups: ≤3 years old (infant group; 102 cases), 3–7 years old (preschool group; 988 cases), and 7–14 years old (school-age group; 1,226 cases). There were 102 cases in the group, among which the top five most common inhalant allergens were *D. pteronyssinus*, *B. tropicalis*, *D. farinae*, dog hair, and cockroach. The top five most common ingestion allergens were milk, egg white, beef/lamb, shrimp/crab, and egg yolk. Among the 988 preschool group cases, 627 cases were attributed to *D. pteronyssinus*, 568 cases to *B. tropicalis*, 542 cases to *D. farinae*, 267 cases to cockroach, and 252 cases to dog hair. The top five most common ingestion allergens were milk, egg white, shrimp/crab, beef/lamb, and egg yolk. There were 1,226 cases of school-age group, with 999 cases attributed to *D. pteronyssinus*, 869 cases to *B. tropicalis*, 943 cases to *D. farinae*, 321 cases to dog hair, and 317 cases to cockroach; The top five most common ingestion allergens were milk, beef/lamb, shrimp/crab, egg white, and egg yolk. There were significant differences in allergens among age classes, including *D. pteronyssinus*, *D. farinae*, *B. tropicalis*, cat hair, Quinoa, smoke aspergillus, and Candida egg white, and milk, whereas no significant differences were observed in the other allergens (Table 2).

### Distribution of positive degree of allergens in children with AR

Among the inhaled allergens of children with AR aged 1–14 years, the top three *D. pteronyssinus* and *D. farinaes* were mainly of level 3 or above, 50.9% (1,180 cases) of *D. pteronyssinus* were ≥3, 47.9% (1,109 cases) of *D. farinaes* ≥3, 23.3% (539 cases) of *B. tropicalis* ≥3. The main allergens were level 2, and the other allergens were level 1 and 2. Among the allergens of children with AR aged 1–14 years, 7.9% (183 cases) had milk level ≥3, 4.7% (108 cases) had shrimp/crab level ≥3, and other allergens were mainly level 1 and 2, as shown in Table 3.

**Table 3:** Grade distribution of different inhalation and ingestive allergens in children with AR.

Allergen	Level 1	Level 2	Level 3	Level 4	Level 5	Level 6	Total
*D. pteronyssinus*	155	339	567	374	84	155	1,674
*D. farinae*	96	315	678	311	62	58	1,520
*B. tropicalis*	330	608	327	108	39	65	1,477
Cat hai	92	160	97	31	4	5	389
Dog hair	268	276	48	6	0	4	602
Cockroach	318	266	16	2	0	0	602
Silk	92	80	5	1	0	0	178
Short ragweed	317	148	8	0	0	0	473
Artemisia pollen	40	14	2	0	2	0	58
Scandent hop	94	38	6	1	0	1	140
Quinoa	268	116	8	0	0	0	392
Juniper	72	47	6	0	0	0	125
Sycamores	232	80	4	0	0	0	316
Willow trees	159	44	5	0	0	0	208
Bluegrass	148	33	5	0	0	0	186
Acer negundo	126	45	4	0	0	0	175
Smoke aspergillus	97	51	6	4	0	0	158
Candida	101	121	26	7	2	1	258
Egg yolk	235	89	5	0	0	0	329
Egg white	305	207	28	3	0	0	543
Milk	291	637	183	0	0	0	1,111
Peanut/Soybean	244	70	7	0	0	1	322
Sesame	64	11	0	0	0	0	75
Wheat/Buckwheat	176	69	7	0	0	0	252
Cashew nuts etc	53	15	2	0	0	0	70
Beef/Mutton	251	162	8	0	0	1	422
Fish	149	42	2	0	0	0	193
Shrimp/Crab	165	136	44	18	12	50	425
Peach/Apple	100	58	2	0	0	0	160

### Seasonal distribution of positive allergens in children with AR

**Figure 3:**
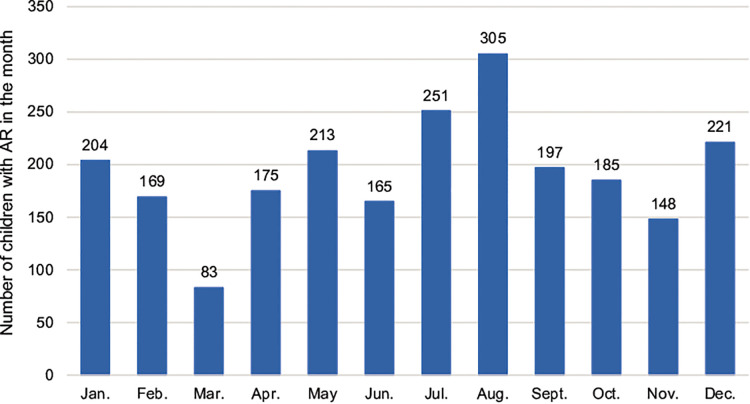
Distribution of cases of children with AR in different months. The abscissa represents different months, and the ordinate represents the number of children with AR.

**Figure 4:**
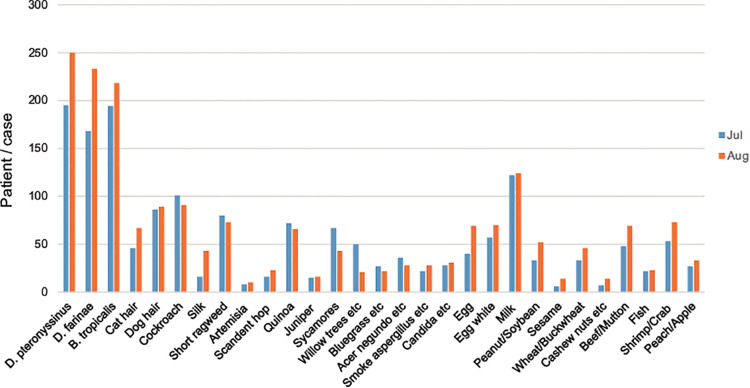
Distribution of allergens in children with AR in July and August. The abscissa represents different allergens, and the ordinate represents the number of children with AR. The blue color stands for July, and the orange color stands for August.

As shown in Figures 3, 4, the peak period of AR children's visits in Shenzhen was concentrated in July and August, with the largest number of visits in August, and relatively few visits in March and November. Most children with AR visited hospitals in summer. In addition, the allergens *D. pteronyssinus*, *B. tropicalis*, *D. farinae*, and milk in children with AR were generally increasing (Figures 3, 4).

## Discussion

### Distribution of allergens

Our study showed that the most common allergens were *D. pteronyssinus, D. farinae,* and *B. tropicalis*. Milk, egg white, and shrimp/crab were the most common ingestion allergens. A previous study found that atopy and allergic sensitization to *D. pteronyssinus* and *D. farinae* were more common in Guangzhou than in Beijing (17), which is in line with the results of the present study. In the past, ingestion allergens in children with AR were frequently ignored; recently, however, it was found that the symptoms of AR may be aggravated by ingestion allergens (7). Inhaled allergens were mainly *D. pteronyssinus* and *D. farinae*; the reason may be that Shenzhen area experiences a subtropical monsoon climate, which is warm and humid, with long summers, short winters, and long rainy seasons, which is conducive to the propagation of mites and cockroaches. In particular, the *B. tropicalis* is an endemic allergen in southern China, which is in line with the geographical characteristics of Shenzhen. Ingestion allergens are mainly milk, egg white, and shrimp/crab. Shenzhen has a high average living standard, and its residents prefer nutritious foods such as milk and egg white, whereas the Pearl River Delta region is rich in aquatic resources, thus seafood allergens such as crab and shrimp are important parts of the local diet and thus act as allergens more frequently. This suggests that the sensitization of ingested allergens is closely related to local economic level and dietary habits. At the same time, the results showed that 1,337 cases of children with allergies (aspiration and ingestion) were positive for five or more allergens: 336 patients were positive for four allergens, 337 patients were positive for three allergens, 222 patients were positive for two allergens, and 84 patients were positive for one single allergen. Since the number of AR children positive for multiple allergens were more than those positive for single allergen, we deduced that allergies may be a kind of constitution. Few AR patients are allergic to one single allergen, and most patients are allergic to multiple allergens. Patients with allergies to mites, in particular, are generally allergic to one mite and show positive reactions to other mite allergens, but the severity of the reactions is different because the main determinants of mite allergens differ between species (18).

### Difference of distribution of allergens between male and female participants

The results of this study showed that the common inhalation allergens of 2,316 children with AR aged 1–14 years were *D. pteronyssinus*, *D. farinae*, and *B. tropicalis*. The most common ingestion allergens of male children with AR were milk, egg white, and shrimp/crab. The most common inhalation allergens in 836 female children with AR were *D. pteronyssinus*, *B. tropicalis*, and *D. farinae*. The most common ingestion allergens were milk, egg white, and beef/mutton. We found that the positive rate of serum sIgE in children with AR was higher in boys than in girls, and it has been reported that the gender difference in the positive rate of allergen sIgE may be related to different inflammatory pathways in different sexes (19). Studies from other countries reported that the prevalence of AR was higher in males than in females, and nasal congestion was the main symptom in males and runny nose and nasal itching in females (20). The positive rate of serum sIgE in male children with AR is higher than that in female children, indicating that boys are more likely to suffer from allergic diseases, which may be related to the regulation of estrogen on the immune system; however, the underlying mechanisms remain to be elucidated (21). The study showed that the main different allergens between different gender of children in Shenzhen region were *D. farinaes*, and boys with AR exhibited significantly higher inhaled allergen positive rates than girls with AR, probably due to different preferences of toys. There is a dose-dependent relationship between the exposure concentration of *D. farinaes* in the environment and the symptoms of AR. Reducing the concentration of *D. farinaes* in the environment can help reduce the incidence of AR in children (22). Therefore, it is necessary to pay more attention to the sensitization of male patients to inhalation allergens in clinical practice. Among the ingestion allergens, only milk and shrimp/crab showed statistical differences between genders, which was firstly related to similar dietary habits of children, and secondly was related to intestinal immune functions of children.

### Distribution of allergens in different age–groups

Different age groups exhibited different allergens. The prevalence of AR increased with age, and the number of children with AR in the school-age group was the highest. Previous studies have shown that AR increases from preschool children and peaks at age 17–18 years (22). Some studies have reported that age is a protective factor for allergic diseases, and the positive rate of allergens gradually decreases with age (23–25). In the three age groups of the present study, the top three inhaled allergens were *D. pteronyssinus*, *D. farinae*, and *B. tropicalis*, while the top three ingestion allergens were milk, egg white, beef/mutton, and shrimp/crab, respectively. The main allergen thus differed between age groups. We speculated that the inhaled allergens of mites are more important for the Shenzhen region with regard to climate and environmental characteristics favoring mite propagation, and mites are typically most abundant in indoor areas, with textiles and objects such as quilts, bed sheets, pillows, cloth sofas, carpets, and stuffed toys being preferred mite hiding places, which are typically present in the main activity areas of children. With increasing age, the range and areas of children's' activities change, and the overlap of allergens caused by seasonal changes may be the reason for the largest number of AR patients in the school group. The order of the ingestion allergen spectrum differed between age groups, which may be due to changes in dietary habits and dietary structure. Overall, avoiding *D. farinae* and improving the family environment is particularly important. With regard to ingestion allergens, parents should be familiar with the most common ingestion allergens in children and should prevent ingestion of allergens by the children, i.e., the children's diet should be monitored and adjusted, and alternatives to the allergenic food sources should be provided.

### Distribution of positive degree of allergies in AR patients

Children with AR showed different severities of responses to inhaled and ingestive allergens. The three common inhaled allergens were *D. pteronyssinus*, *D. farinaes*, and *B. tropicalis*; the frequency of allergies to *D. pteronyssinus* and *D. farinaes* was obviously higher than those to *B. tropicalis*. In addition, the severity of AR children positive for *B. tropicaliss* was mainly on levels 1, 2, 3, or lower. First, this may be affected by the climate in Shenzhen. Exposure to mites at high abundances is typically long-lasting, especially during the rainy season. Previous studies have shown that the time of exposure to allergens is the key factor of AR (26, 27). Second, the prevalence of AR in children may be related to increased exposure to indoor allergens, especially *D. farinae*, due to indoor temperatures, humidity, and living environments (28).

### Seasonal distribution of allergens in children with AR

Season is one of the main factors affecting the occurrence of AR. The results showed that the peak visits by children with AR children were in July and August, and the number of visits was relatively small in March and November. Shenzhen is a coastal city in southern China, adjacent to Hong Kong. It has a subtropical monsoon climate with long summers and short winters, which is generally mild with abundant sunshine and rainfall. According to the Shenzhen Meteorological Bureau report released in 2021, the four seasons of Shenzhen in recent years are roughly divided into spring (early February to mid-April), summer (late April to early November), autumn (early November to mid-January of the following year), and winter (mid-January to early February). The present results showed that AR was most common in July and August, which in Shenzhen corresponds to warm moist air flows of the summer, with high temperatures and high humidity. These conditions are conducive to the propagation of mites and cockroaches, thus markedly increasing the abundances of mites. In addition to the increase of mite exposure, this season also coincides with the pollen peak. For AR patients in the summer, short ragweed、quinoa, Amaranthus retroflexus are important allergens, in addition to mites; short ragweed flowers from July to September, quinoa flowers for 5–10 months, and Amaranthus retroflexus flowers from July to August. In line with our results and additive effects of allergens, this may explain the observed high prevalence of AR in children during summer. The number of hospitalized AR children is relatively small in March and November. Considering the climate change, dropping temperature, and humidity at these times, we hypothesized that the exposure chance and concentration of mites have decreased, explaining the relieving symptoms in the AR children.

## Data Availability

The raw data supporting the conclusions of this article will be made available by the authors, without undue reservation.
